# Dynamic Production Control and Optimization under Low Carbon Economy considering Delivery Time Demand

**DOI:** 10.1155/2022/4535383

**Published:** 2022-05-21

**Authors:** Ming Liu, Ting Yang, Qingjun Li

**Affiliations:** ^1^School of Economics and Management, Weifang University, Weifang 261061, China; ^2^Tianrui Maglev Industrial College, Weifang University, Weifang 261061, China

## Abstract

Production control plays an important role in the efficient use of production resources. To promote energy-saving and environmental protection, low carbon emissions, and sustainable development of enterprises, it is necessary to explore the production control scheme that facilitates the manufacturing of low-cost products. However, the existing studies have not incorporated expected delivery time into the preparation of production control scheme. With the purpose of improving the low carbon economy, this paper integrates the quantitative data of the dynamic production control process to reduce the overall cost and solves the problems of collaborative operation between dynamic production control and delivery time demand, aiming to promote the green and sustainable development of production control technologies of the manufacturing industry. To solve the problems more scientifically, the following works were done: firstly, a dynamic production control model was established under the constraint of low carbon emissions. Next, the corporate manufacturing information was synthetized with the data on production, logistics, and delivery, and a collaborative optimization model was established for the dynamic production control and delivery time demand under low carbon economy. The effectiveness of our model was demonstrated through experiments.

## 1. Introduction

With the advancement of Industry 4.0 and Made in China 2025, the green manufacturing of low carbon products has become an irreversible trend [1–6]. Production control plays an important role in the efficient use of production resources [7–12]. If the production control mode can timely satisfy the consumer demand of delivery time, it is possible to collaboratively optimize dynamic production control and delivery time demand [13–18]. The manufacturing industry faces the urgent need to save energy, protect the environment, and cut down carbon emissions. To ensure sustainable development, manufacturers must explore the production control scheme that facilitates the manufacturing of low-cost products [19–22].

Most of the traditional analyses based on the production control system assume that the system is linear, the time to deliver the products to the final consumers is negligible, or the delivery time is a proxy variable. Lin et al. [23] combined the difference of delivery lead time, supplier's capacity availability, and difference of inventory at the order decoupling point into the performance triangle, and utilized the performance triangle and nonlinear engineering control to evaluate the dynamic production control of automatic train operation system. Dhahri et al. [24] explored the integrated production and delivery control of an unreliable manufacturing system and multiple retailers, optimized the strategy for joint production and delivery control with the minimal total cost, which includes inventory/backlog cost excluding transport cost, and proposed the optimal control strategy by random optimal control and pulse control theories. With the aid of random fluid model, Sadok and Nidhal [25] established the optimal production control with the minimal total cost of inventory, transport, and sales, in light of machine failure, return, remanufactured products, and delivery activities. The control strategy was applied to an optimization algorithm, which optimizes the manufacturing inventory under the constraints of return and delivery time. Takahashi et al. [26] considered a dual-channel supply chain model involving two levels (production and delivery), proposed a novel supply chain storage control strategy, and constructed a new control strategy for the total cost of the two-level dual-channel supply chain. The total cost consists of inventory holding cost, sales loss cost, and production and delivery configuration cost. Finally, the total cost was calculated by Markov analysis, which demonstrates the effectiveness of their control strategy. Hung et al. [27] tracked production progress, control delivery, and satisfying consumer demand by applying the production plan, output-line analysis, and lot tracing system. The system increased the delivery rate by 35% and significantly reduced the time cost of progress control.

In summary, the domestic and foreign studies on production scheduling, especially the production control of manufacturers, have not incorporated expected delivery time into the preparation of production control scheme. The ensuing product retention is very likely to cause an increase in the operational cost of production control and dampen consumer satisfaction. With the development of the Internet and mobile payment, it is increasingly easy for consumers to communicate with enterprises. If the two parties cannot coordinate the delivery time well, they will see a rise in all sorts of costs. Therefore, this paper investigates the dynamic production control and optimization under low carbon economy considering delivery time demand.  Section 2 introduces the constraint of low carbon emissions to the traditional dynamic production control research and constructs a dynamic production control model under the constraint of low carbon emissions. Focusing on the machines, parts, and consumers in production process, Section 3 synthetizes the corporate manufacturing information with the data on production, logistics, and delivery, establishes a collaborative optimization model for the dynamic production control and delivery time demand under low carbon economy, and explains the flow of dynamic production control and optimization under low carbon economy considering delivery time demand. The effectiveness of our model was demonstrated through experiments.

## 2. Dynamic Production Control Model under the Constraint of Low Carbon Emissions

**Figure 1:**
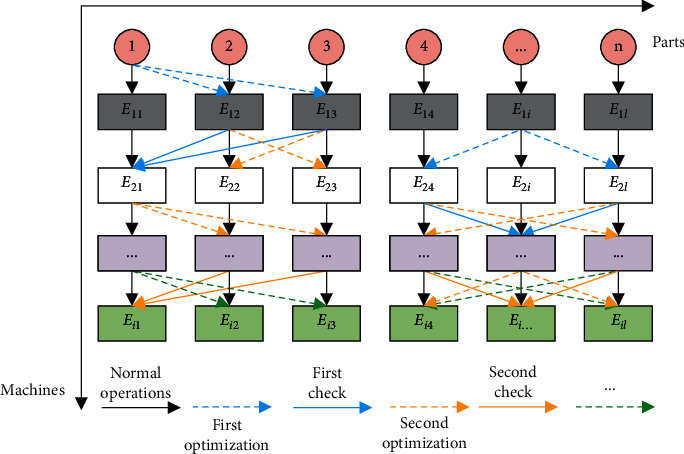
Parts processing under dynamic production control.

This paper investigates the dynamic production control and optimization under low carbon economy considering delivery time demand. During the investigation, the first step is to build a dynamic production control model under the constraint of low carbon emissions. The model was established by adding the constraint of low carbon emissions to the traditional research into dynamic production control. Besides, the carbon emissions of machines were configured under different conditions, and the time factors, e.g., the loading/unloading time of parts or materials, that affect machine operation were taken into account.  Figure 1 shows the execution of each operation for parts processing on the corresponding machine under dynamic production control. The optimization mechanism and verification mechanism run multiple times, rather than only two rounds.

The model variables and parameters are defined as follows: *m* is the total number of parts; *n* is the total number of machines; *f* is the serial number of machines; *i* is the serial number of parts; *j* is the serial number of operations; *P*_*ij*_ is operation *j* of part *i*; *P*_*ij*_^*f*^ indicates that *P*_*ij*_ is executed on machine *f*; *o*_*ij*_^*f*^ is the loading time of *P*_*ij*_ on machine *f*; *τ*_*ij*_^*f*^ is the processing time of *P*_*ij*_ on machine *f*; *v*_*ij*_^*f*^ is the unloading time of *P*_*ij*_ on machine *f*; *τ*_*w*_^*f*^ is the start time of machine *f*; *τ*_*l*_^*f*^ is the no-load time of machine *f*; *τ*_*e*_^*f*^ is the time of machine *f* processing parts at the speed *U*_*E*_; *U*_*E*_ is the processing speed of an operation on the corresponding machine; *R*^*w*^ is the mean carbon emissions of machines during the start time; *R*^*l*^ is the mean carbon emissions of machines in no-load state; *R*^*E*^ is the mean carbon emissions of machines at different speeds; *SH*_max_ is the makespan; *R* is the total carbon emissions of the production process. The operation execution state can be characterized by the following binary function:

(1) $$\begin{array}{c} a_{ij}^{f}=\left\{\begin{array}{cc} 1, & P_{ij}\text{is executed on machinef,}\\ 0, & \text{Otherwise,} \end{array}\right)\\ S_{ijhk}^{f}=\left\{\begin{array}{cc} 1, & P_{ij}\text{is executed on machinefprior to }P_{hl},\\ 0, & \text{Otherwise.} \end{array}\right) \end{array}$$

In the dynamic production control under the constraint of low carbon emissions, low carbon becomes an objective alongside traditional objectives like makespan and machine load. Let *SH*_*i*_ be the completion time of the *i*-th processing part. The objective function of minimal makespan can be expressed as

(2) $$\begin{array}{c} \text{min}SH_{\text{max}}=\text{min}\left(\text{max}\left\{SH_{i}|i=1,2,\ldots ,m\right\}\right). \end{array}$$

The maximum machine load can be expressed as

(3) $$\begin{array}{c} g_{2}=\text{min}Q_{n}=\text{min}\left(\text{max}\left\{\sum_{i=1}^{m}\sum_{j=1}^{m_{i}}\tau_{ijf}\right\}\right). \end{array}$$

The total machine load can be expressed as

(4) $$\begin{array}{c} g_{3}=\text{min}Q_{\psi }=\text{min}\left(\sum_{i=1}^{m}\sum_{j=1}^{m_{i}}\sum_{f=1}^{n}a_{ijf}\tau_{ijf}\right). \end{array}$$

The objective function of minimal total carbon emissions can be expressed as

(5) $$\begin{array}{c} \text{min}R=\overset{n}{\underset{f=1}{\sum }}R^{w}\cdot \tau_{w}^{f}+\overset{n}{\underset{f=1}{\sum }}R^{l}\cdot \tau_{l}^{f}+\overset{n}{\underset{f=1}{\sum }}R^{e}\cdot \tau_{e}^{f}. \end{array}$$

The processing sequence of the operations of the parts must satisfy the following constraint:

(6) $$\begin{array}{c} v_{ij}\leq o_{i\left(j+1\right)}. \end{array}$$

**Figure 2:**
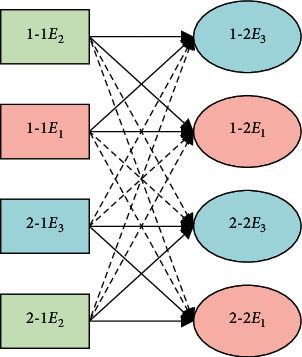
Example of machine allocation under dynamic production control.

Figure 2 gives an example of machine allocation under dynamic production control. Each operation of parts can only be executed on one of the machines:

(7) $$\begin{array}{c} \overset{n}{\underset{f=1}{\sum }}a_{ij}^{f}=1. \end{array}$$

The sequence of two jobs on a machine must satisfy the following constraint:

(8) $$\begin{array}{c} \text{if }S_{ijhk}^{f}=1,\;\text{then }v_{ij}^{f}\leq o_{hk}^{f}. \end{array}$$

## 3. Collaborative Optimization Model

**Figure 3:**
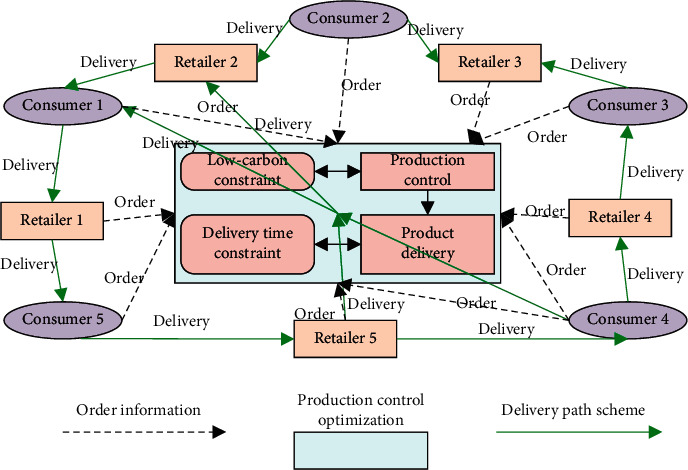
Architecture of the collaborative optimization model for dynamic production control and delivery time demand under low carbon economy.

Considering the machines, parts, and consumers of the production process, this paper extends the dynamic production control model under the constraint of low carbon emissions into a collaborative optimization model for dynamic production control and delivery time demand under low carbon economy. The model has four optimization objectives: production cost, machine maintenance cost, penalty cost, and total cost. Dynamic production control and delivery time demand were optimized collaboratively by synthetizing the corporate manufacturing information with the data on production, logistics, and delivery. To solve the collaborative optimization problem under low carbon economy, it is necessary to address the following issues simultaneously: select the right machine for parts or materials, arrange the proper sequence of machines for parts, and realize low carbon production.  Figure 3 shows the architecture of the collaborative optimization model for dynamic production control and delivery time demand under low carbon economy. The production control and delivery time under low carbon economy have an additional constraint, namely, low carbon economy, compared with that under the general carbon economy.

Let *FS*_*n*_, *FS*_*o*_, *FS*_*W*_, and *FS*_*or*_ be the machine maintenance cost, production cost, carbon cost, and penalty cost, respectively. Then, the objective function of the collaborative optimization model for dynamic production control and delivery time demand can be established as

(9) $$\begin{array}{c} \text{min}FS=\text{min}\left(FS_{n}+FS_{o}+FS_{W}+FS_{or}\right). \end{array}$$

**Figure 4:**
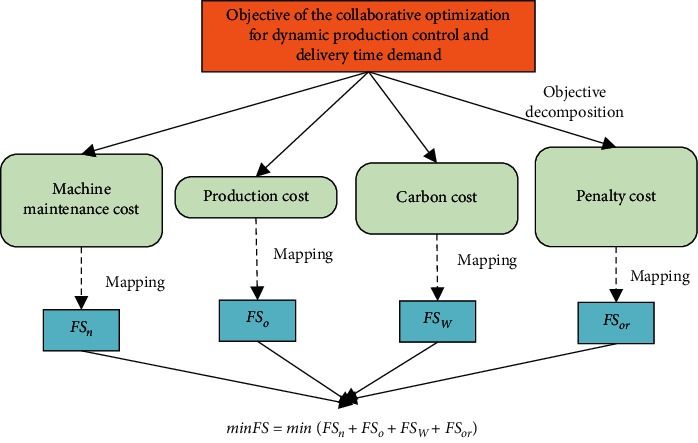
Decomposition of the objective function.

Figure 4 breaks down the objective function. Each of the four costs is detailed as follows. To accurately estimate the machine maintenance cost, it is important to summarize the failure law of machines and quantify the failure uncertainty. Suppose machine failures obey the Weibull distribution with the shape parameter *α* and scale parameter *δ*. Then, the failure rate of machines can be described as

(10) $$\begin{array}{c} \mu \left(\tau \right)=\frac{\alpha }{\delta }{\left(\frac{\tau }{\delta }\right)}^{\alpha -1}e^{-{\left(\tau /\delta \right)}^{\alpha }}. \end{array}$$

Based on formula (10), the expected number of failures *ET*(*t*) of machines in production cycle *t* can be calculated by

(11) $$\begin{array}{c} ET\left(t\right)=\int_{0}^{t}\mu \left(\tau \right)d\tau =\int_{0}^{t}\frac{\alpha }{\delta }{\left(\frac{\tau }{\delta }\right)}^{\alpha -1}e^{-{\left(\tau /\delta \right)}^{\alpha }}d\tau . \end{array}$$

Let *τ*^*∗*^ be the execution time of the maintenance strategy, and *τ*_*on*_ and *τ*_*ns*_ be the execution time *τ*^*∗*^ of preventive maintenance and minor maintenance, respectively. Then, the mean failure time of machines can be calculated by

(12) $$\begin{array}{c} \psi_{1}={\sum \tau }^{*}\int_{0}^{t}\frac{\alpha }{\delta }{\left(\frac{\tau }{\delta }\right)}^{\alpha -1}e^{-{\left(\tau /\delta \right)}^{\beta }}d\tau . \end{array}$$

Let *FS*_*vn*_ and *FS*_*vo*_ be the maintenance cost and processing cost per unit of time, respectively. Then, the machine maintenance cost during dynamic production control can be expressed as

(13) $$\begin{array}{c} FS_{n}=FS_{vn}\cdot \tau_{\text{no}}+FS_{vo}\cdot \psi_{1}. \end{array}$$

If the consumer demand for delivery time is not satisfied, the enterprise and consumers may suffer capital loss, and the enterprise may lose potential consumers. Let *φ*^*n*^ and *φ*^*m*^ be the earliest and latest delivery time satisfying delivery time demand, respectively; *φ*^*r*^ and *φ*^*l*^ be the actual earliest and latest delivery time, respectively; and *ω* and *ε* be the early penalty factor and late penalty factor, which, respectively, stand for the penalty cost against early delivery and that against late delivery.

If the delivery time falls in [*φ*^*r′*^, *φ*^*n*^], then the delivery is completed early and should subject to an early penalty cost; if the delivery time falls in [*φ*^*r′*^, *φ*^*r*^], then the delivery is completed too early and should subject to a high early penalty cost. To reduce the loss induced by the lack of collaboration between the production plan and the delivery time demand, this paper proposes a penalty factor about the penalty cost against the failure to meet the client's delivery time demand and establishes a penalty cost function constrained by the delivery time window. If the delivery time falls in [*φ*^*m′*^, *φ*^*k*^], then the delivery is completed late and should subject to a late penalty cost; if the delivery time falls in [*φ*^*k′*^, *φ*^*k*^], then the delivery is completed too late and should subject to a high late penalty cost. The penalty cost function can be expressed as

(14) $$\begin{array}{c} FS_{or}=\left\{\begin{array}{c} \frac{\phi^{n}-\tau^{\prime }}{\phi^{m}-\phi^{n}}\cdot \omega^{2},\tau^{\prime }\in \left[\phi^{r},\phi^{r^{\prime }}\right]\\ \frac{\phi^{n}-\tau^{\prime }}{\phi^{m}-\phi^{n}}\cdot \omega ,\tau^{\prime }\in \left[\phi^{r^{\prime }},\phi^{n}\right]\\ 0,\tau^{\prime }\in \left[\phi^{n},\phi^{m}\right]\\ \frac{\phi^{n}-\tau^{\prime }}{\phi^{m}-\phi^{n}}\cdot \varepsilon ,\tau^{\prime }\in \left[\phi^{m},\phi^{k^{\prime }}\right]\\ \frac{\phi^{n}-\tau^{\prime }}{\phi^{m}-\phi^{n}}\cdot \varepsilon^{2},\tau^{\prime }\in \left[\phi^{k^{\prime }},\phi^{k}\right] \end{array}|.\right. \end{array}$$

During the production, the machines consume lots of energy, which supports machine operation and dissipates in the form of carbon emissions. Here, the carbon emissions of machines are estimated based on the different machine states. Let *GV*_*l*_ be the standby power of machine *l* and *ψ*_*l*_ be the standby time of machine *l*. Then, the energy consumption *W*_1_ of a machine in standby state can be calculated by

(15) $$\begin{array}{c} W_{1}=\sum GV_{l}\psi_{l}. \end{array}$$

Let *x* and *y* be the start time and end time of machine operation, respectively; *ψ*_*l*1_ be the time it takes for a machine from start to stable operation; *ψ*_*l*2_ be the time it takes for a machine from shutdown to stop; and *GV*_*l*_(*τ*) be the input power of machine *l* at time *τ*. Then, the energy consumption *W*_2_ of a machine in start and shutdown period can be calculated by

(16) $$\begin{array}{c} W_{2}=\int_{x}^{x+\psi_{l1}}GV_{l}\left(\tau \right)d\tau +\int_{y-\psi_{l2}}^{y}GV_{l}\left(\tau \right)d\tau . \end{array}$$

Let *GV*′_*l*_ be the operating power of machine *l* and *τ* be the processing time of that machine. Then, the energy consumption *W*_3_ of a machine in processing state can be calculated by

(17) $$\begin{array}{c} W_{3}=\sum GV_{l}^{\prime }\tau . \end{array}$$

Let *GV*_*l*3_ be the no-load operating power of machine *l* and *ψ*_*l*3_ be the no-load operating time of machine *l*. Then, the energy consumption *W*_4_ of a machine in no-load operation can be calculated by

(18) $$\begin{array}{c} W_{4}=\sum GV_{l3}\psi_{l3}. \end{array}$$

Let *σ* be the conversion factor from each unit of energy consumption to carbon emissions. Then, the carbon emissions in the processing process can be calculated by

(19) $$\begin{array}{c} W_{c}=\sigma \left(W_{1}+W_{2}+W_{3}+W_{4}\right). \end{array}$$

Let *FS*_*vr*_ be the unit cost of energy consumption. The carbon cost in the corresponding production stage can be calculated by

(20) $$\begin{array}{c} FS_{W}=FS_{vr}\left(W_{1}+W_{2}+W_{3}+W_{4}\right)\cdot \sigma . \end{array}$$

It is assumed that the production cost *FS*_*o*_ is positively proportional to processing time. Let *τ*_*i*_ be the completion time of part *i* and *FS*_*vo*_ be the processing cost per unit time. Then, the production cost can be expressed as

(21) $$\begin{array}{c} FS_{o}=FS_{vo}\cdot \tau_{i}. \end{array}$$

Considering delivery time demand, the collaborative optimization model for dynamic production control and optimization under low carbon economy can be expressed as

(22) $$\begin{array}{c} M=\text{min}\left(FS_{vn}\cdot \tau_{\text{on}}+FS_{vo}\cdot \psi_{1}+FS_{\text{or}}+FS_{vr}\left(W_{1}+W_{2}+W_{3}+W_{4}\right)\cdot \sigma +FS_{vo}\cdot \tau_{i}\right). \end{array}$$

**Figure 5:**
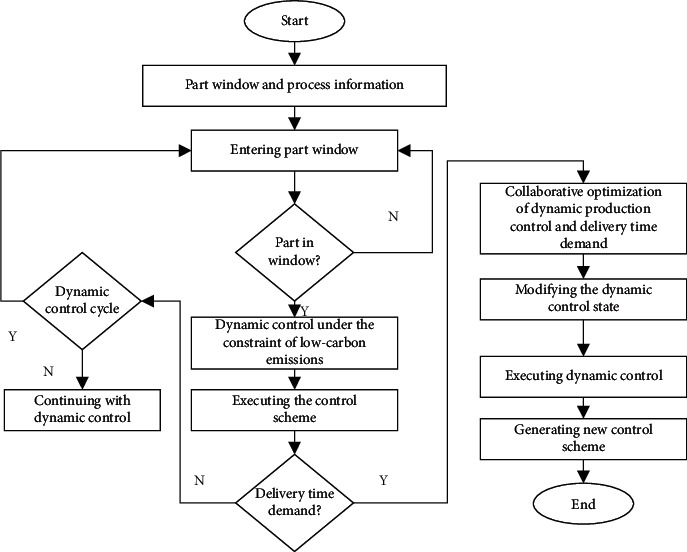
Flow of dynamic production control and optimization under low carbon economy considering delivery time demand.

Figure 5 illustrates the flow of dynamic production control and optimization under low carbon economy considering delivery time demand. Before starting the calculation of entering part window, the number of maximum iterations should be set to 100, and the loss threshold should be set as 0.001.

## 4. Experiments and Result Analysis

**Figure 6:**
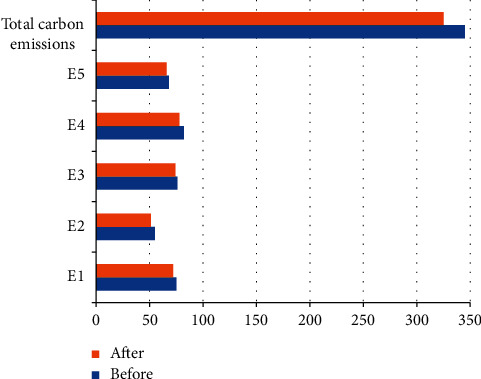
Total carbon emissions before and after adjusting noncritical operations.

**Figure 7:**
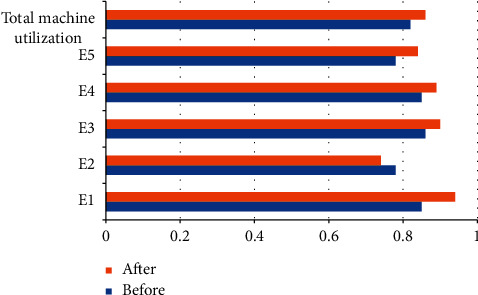
Total machine utilization before and after adjusting noncritical operations.

The proposed model was solved, and the optimal solution set for production control scheme was screened (Table 1). After dynamic production control, the makespan did not change significantly, the total carbon emissions through the production decreased obviously, and the machine utilization increased prominently. Figures 6 and 7 compare the total carbon emissions and total machine utilization before and after dynamic production control, respectively.

The proposed collaborative optimization model and algorithm were applied to solve the actual completion time.  Table 2 compares the results with the delivery time expected by consumers.

In Table 2, the completion time of the last operation is taken as the completion time of the product to be delivered. It can be observed that the proposed collaborative optimization scheme basically satisfied consumer demand for delivery time. The delivery of products 1, 2, 3, and 5 all met the expected delivery time; Product 4 was delivered 7.6 min earlier than the earliest time expected by consumers; Product 6 was delivered 18.4 min later than the latest time expected by consumers.

The above comparison shows that the product manufacturing can be completed early, if the production control fully considers consumer demand for delivery time; otherwise, the product manufacturing is generally completed late. In addition, the machines are not well utilized, when the production control scheme overlooks delivery time demand.  Table 3 compares the completion time, satisfaction of delivery time demand, and machine utilization before and after the consideration of delivery time demand.

As shown in Table 3, when delivery time demand was not considered, the machine utilization results were relatively small in the optimal solution set of the production control scheme: *E*_1_ 62.58%, *E*_2_ 82.58%, *E*_3_ 75.18%, *E*_4_ 52.48%, *E*_5_ 78.42%, and *E*_6_ 74.15%. When delivery time demand was considered, the machine utilization results were *E*_1_ 55.48%, *E*_2_ 65.29%, *E*_3_ 57.26%, *E*_4_ 45.75%, *E*_5_ 68.29%, and *E*_6_ 85.24%.

The numerical results without considering delivery time demand were compared with those obtained by our collaborative optimization model, indicating that our model achieved significant improving effects on cost and consumer satisfaction.  Table 4 compares the various cost items, including machine maintenance cost, carbon cost, and penalty cost.

As shown in Table 4, after considering delivery time demand, our collaborative optimization model not only met the consumer demand of delivery time, but also improved machine utilization. Therefore, our model has an advantage in solving similar problems. Compared with the scheme without considering delivery time demand, our optimal production control scheme reduced the machine maintenance cost, carbon cost, penalty cost, production cost, and total cost by 26.45%, 1.45%, 23.95%, 14.39%, and 86.39%, respectively. Overall, our model realized higher production efficiency and lower production cost than the model that does not consider delivery time demand.

## 5. Conclusions

This paper investigates dynamic production control and optimization under low carbon economy, considering delivery time demand. Specifically, the constraint of low carbon emissions was introduced to the traditional research into dynamic production control, and a dynamic production control model was established under such a constraint. Next, the manufacturing information was integrated with the data on production, logistics, and delivery, in light of the machines, parts, and consumers in the production process. On this basis, a collaborative optimization model was constructed for dynamic production control and delivery time demand under low carbon economy, and the flow was specified for dynamic production control and optimization under low carbon economy, considering delivery time demand. Through experiments, the optimal solution set of production control scheme was screened, and the carbon emissions and machine utilization before critical process adjustment were compared with those after the adjustment. The comparison shows that, after the adjustment, the total carbon emissions were significantly suppressed, and machine utilization was clearly improved. In addition, the actual completion time was compared with delivery time demand, and the analysis results before and after considering delivery time demand were contrasted with each other. Finally, the cost items of our model were compared with those of the model without considering delivery time demand. It is concluded that our model realized higher production efficiency and lower production cost than the model that does not consider delivery time demand.

## Figures and Tables

**Table 1:** Screened optimal solution set for production control scheme.

Production control scheme	1	2	3	4	5	6	7
Makespan	65	63	62	65	62	68	82
Maximum machine load	56	52	58	54	55	52	56
Total machine load	355	362	345	358	345	342	362
Total carbon emissions	582	595	526.3	542.5	528.4	516.2	504.2

**Table 2:** Actual completion time vs. delivery time demand.

Operation		Product 1	Product 2	Product 3	Product 4	Product 5	Product 6
		1–6	2–6	3–6	4–6	5–6	6–6
Actual completion time		335.2	375.1	412.5	352.4	405.2	368.4
Accelerated penalty time window	Earliest	340	360	395	355	390	330
	Latest	370	390	425	385	425	355
Delivery time demand	Earliest	350	370	400	360	400	340
	Latest	360	385	420	380	415	350
Demand satisfied? (*T*/*F*)		*T*	*T*	*T*	*F*	*T*	*F*
Deviation		0	0	0	7.6	0	−18.4

**Table 3:** Results before and after considering delivery time demand.

Product Operation		Product 1	Product 2	Product 3	Product 4	Product 5	Product 6
		1–6	2–6	3–6	4–6	5–6	6–6
Considering delivery time demand	Completion time	352.6	415.8	405.1	338.6	422.7	362.5
	Demand satisfied? (*T*/*F*)	*T*	*T*	*F*	*T*	*T*	*F*
	Machine utilization	*E*_1_ 62.58%	*E*_2_ 82.58%	*E*_3_ 75.18%	*E*_4_ 52.48%	*E*_5_ 78.42%	*E*_6_ 74.15%

Without considering delivery time demand	Completion time	352.4	475.2	428.1	462.7	375.8	395.2
	Demand satisfied? (*T*/*F*)	*F*	*F*	*T*	*F*	*T*	*T*
	Machine utilization	*E*_1_ 55.48%	*E*_2_ 65.29%	*E*_3_ 57.26%	*E*_4_ 45.75%	*E*_5_ 68.29%	*E*_6_ 85.24%

**Table 4:** Comparison of cost items.

Cost item		Machine maintenance cost	Carbon cost	Penalty cost	Production cost	Total cost
Optimal results	Without considering delivery time demand	75.14	251.82	322.58	524.15	1173.69
	Our model	55.26	248.15	245.29	448.72	972.85

Optimization rate		26.45	1.45	23.95	14.39	86.39

## Data Availability

The data used to support the findings of this study are available from the corresponding author upon request.
